# Royal Academy of Medicine in Ireland

**Published:** 1888-02

**Authors:** 


### ROYAL ACADEMY OF MEDICINE IN IRELAND.

### Pathological Section—Meeting, Friday, November 4, 1887.

The President, Dr. C. B. Ball, in the Chair.

The Session for 1887-88 of the Academy was opened at the Royal College of Surgeons in Dublin, by the meeting of the Pathological Section.

The President delivered an address on Tetanus, after which Dr. Finny exhibited a specimen of Hour-glass Contraction of the Stomach, which was the seat of several ulcers.

The following were the clinical history and morbid appearances: Mary M'G., a domestic servant, æt.29, was admitted into Sir Patrick Dun's Hospital on May 13, 1887, in a state of collapse and in great pain, which was referred to the lower part of the abdomen. After reaction had set in, under appropriate treatment, she vomited once, and the rejected matter consisted of tea and milk, but contained no blood. The urine was drawn off and found to be free from albumen and indican. All her symptoms were those of acute peritonitis from perforation. Death ensued eight hours after admission. Her history was that she had enjoyed good health up to three weeks before admission, but beyond some slight dyspepsia, she was able to continue her duties as a domestic servant up to the day previous to her admission. The post-mortem examination, made thirty-six hours after death, displayed acute general peritonitis, with flaky lymph and sero-purulent fluid, while the stomach was found to be the seat of recent inflammation, as well as of old and firm adhesions to the liver, spleen, pancreas, and diaphragm. At first sight there seemed to be two stomachs, or one with a very dilated duodenum; and this appearance was due to a constriction transverse of the stomach, nearer to the pylorus than to the cardia. On the anterior surface of the cardiac half of the organ, not far from the greater curvature, the origin of the general peritonitis was seen in the existence of a circular, sharply-defined, perforating ulcer, the size of a threepenny piece. On laying open the stomach it was found to have been the seat of several ulcers—(1), that referred to on the anterior wall, which on the mucous aspect measured a sixpence, and had the punched-out characteristic appearance; (2), a firmly cicatrized, circular ulcer on the posterior wall, near the lesser curvature—which was not in a state of activity--whose floor consisted of the pancreas to which it was ultimately adherent; (3), a small ulcer in a stage of activity lower down in the posterior wall; and (4), an oval ulcer extending three-quarter inch from the lesser curvature, transversely to the long axis of the stomach, and in an active stage of destruction of the mucous and part of the muscular coat. This ulcer ran into the circular constriction which divided the stomach into two unequal bags. The constriction, which was half an inch wide, narrowed the organ so as to admit but three fingers, and had all the appearance, from external examination, of the pylorus. The lesser curvature was not dimpled or marked much, but towards it the greater curvature was drawn at this spot, so as to make two bags of the stomach. Its outside was thickened by old lymph, while on the inside the rugæ of the stomach seemed to run into it, and at its cardiac side was the ulcer referred to as No. 4. Dr. Finny discussed the pathological origin of the constriction, and remarked on its want of agreement with the features depended upon by Dr. W. R. Williams as distinctive of congenital hour-glass contraction. He alluded to the rarity of cases of this deformity of the stomach—the greater number having been discovered in the dissecting-rooms. He also laid stress on the following, as exemplified by his specimen--(1), the comparative greater danger of an ulcer situated within the anterior wall; (2), the co-existence of three ulcers in a state of activity; (3), the possibility of relapse; (4), the encouragement to treatment to be derived from the fact that so many ulcers—about half of those recorded—have been discovered in a state of cicatrization.

Dr. MacSwiney asked, Was there any history of the woman having taken food immediately, or soon before, she was attacked with the violent pain and collapse? Very frequently in such cases the rupture occurred in consequence of the stomach being distended with food. He thought Dr. Finny had established that the contraction in this case was pathological.

Dr. Finny, in reply, said there was history of food having been taken immediately before the occurrence of the great pain. The woman had never complained of any pain in the stomach, or other marked gastric symptom, until three weeks before her admission. She was doing her work as a servant when the sudden occurrence of pain obliged her to seek relief. The case was an example of ulcers painlessly eating their way through the walls of the stomach, and ending in perforation at short notice. The woman had vomited before she came into the hospital, and in consequence of the small size of the opening caused by the fatal ulcer nothing was extruded through it.

Dr. Lentaigne communicated a case of Epispadias.

Professor Bennett submitted a specimen from a case of Phlegmasia Alba Dolens.

The patient, a girl, æt. 15, was admitted on the 30th of September, 1886, into Sir Patrick Dun's Hospital, and remained there until the 27th of June, 1887, when she died. When admitted she had phlegmasia alba dolens in the whole of the left leg, which was distended with white œdema. She was extremely emaciated and anæmic, and her œdemic leg was in strong contrast with the rest of her body. There was great difficulty in assigning any cause for the ailment, pregnancy and parturition being entirely out of the question. She suffered from great gastro-intestinal irritation, which was complicated with vomiting and diarrhœa, accompanied with pain. So prominent were these symptoms that at first we thought that the case might be one of typhoid fever, which was even approaching the stage of recovery. The course of the case put that diagnosis out of the question. There was always great abdominal pain and irritation, which could not be controlled by any remedy save the most careful attention to diet, milk being the only thing that the patient could bear. Three weeks after her admission œdema of the right limb commenced, and the condition of the right leg soon became the same as that of the other. Diagnosis then became easy, it being apparent that there was obstruction of the femoral and iliac veins, and that it had extended up the involved vein on the right side. Clinically the case was an extremely easy one to read, and before the swelling of the right limb occurred it was prognosticated that it would occur. The femoral vein could be felt hard and distended on the left side, and when the œdema on the right side occurred the vein on the opposite side was as firm and hard under the finger as a cord. After this, without any material change in the general system, collateral circulation of the venous system of the abdomen began to establish itself, with a reversal of the blood currents, these being directed towards the thorax, instead of the groin. There was then some slight clearing up of œdema, and just a prospect of recovery. Still the gastro-intestinal irritation remained; and then suddenly there came an accession of symptoms, indicating uræmic poisoning. Then there was rapidly developed extreme œdema of the whole body, which pitted rapidly and finally, with uræmic phenomena, death took place. He would now show what they had guessed at as the cause of the disease, but could not distinguish with certainty. There were two great cylindrical patches, close to each other, in the lower portion of the small intestine, of tubercular ulceration of the intestine, with a great series of enlarged glands infiltrated with tubercular matter occupying the mesentery. There was no tuberculosis of the lung; but the gastro-intestinal phenomena appeared to be produced entirely by the condition of the small intestine. The femoral veins and tributaries were occluded. The vena cava inferior was a complete fibrous cord, shrunk and flattened out. When it was traced up between the kidneys there was found a great, firm thrombus, which had already undergone calcification. Then came the most interesting point in the case. On the surface of that calcified thrombus, and projecting up the occluded vein, with its base between the veins of the kidneys, was a fresh, newly-formed thrombus superadded upon it. These thrombi had extended into the renal veins, which were completely occluded in their paths towards the kidneys; and the greater number of the small branches in the substance of the kidneys were also absolutely and entirely occluded. By the second deposit of coagulum on the basis of the old thrombus, and its recent extension into both the renal veins, the fatal issue was brought about, accompanied by the uræmic phenomena of suppression of the urinary secretion, and the rapid development of general anasarca, which was absent in the earlier stages of the case.

Dr. WRIGHT asked, Was the condition of the heart examined?

Dr. DUFFEY said that Trousseau, in his lectures, dicussed the cause of phlegmasia alba dolens, and referred to a case in which there was a swelling of the leg, with a subsequent development of collateral circulation and engagement of the other extremity, attended with intense gastric irritability; and from his knowledge of other cases he predicted the existence of malignant disease of the abdomen, although there was no tumor or other symptom of such disease. Here the disease, though not of a malignant nature, seemed to be very similar to it; and, as far as he understood, the mere pressure of tubercular matter did not appear to have had any affect in causing the obstruction. Trousseau also spoke of extreme anæmia as a cause of phlegmasia dolens. He (Dr. Duffey) had a case in the City of Dublin Hospital of swelling of the leg, in which the only apparent cause of the disease was anæmia. There was a fibrogenetic condition of the blood, to which Trousseau attributed the obstruction of veins.

Professor PURSER said the chief point of interest appeared to be what effect the thrombosis of the renal veins produced on the secretion of urine. The idea generally held was that the quantity of urine secreted depended on the pressure of the blood on the renal capillaries. A remarkable case was recorded of a man in tolerably fair health, in whom an obstruction of the renal veins occurred, and in that case the secretion of urine was increased; but there was a good deal of doubt about the case, and it was important to know from Professor Bennett whether, when the uræmic symptoms commenced, and obstruction of the renal veins, as he believed, occurred, the secretion of urine increased or diminished to any marked degree. It was also important to know whether there was, previous to the occurrence of those symptoms, any albumen in the urine, and whether there was amyloid disease; for gastric irritation was not a common symptom in cases of mere tubercular ulceration of the intestines, particularly where it was so slight as in the present case. It was not improbable that the diarrhœa was due to degeneration of the blood vessels—amyloid degeneration—and, if so, the condition of the kidneys would not have been of such a kind as to make the change in the circulation of any value, one way or the other, as regarded the secretion of urine.

Professor Bennett, in reply, said the heart was healthy; there was nothing whatever in its condition to call for description. There was no evidence of a tumor in the abdomen at any time. The thrombosis commenced apparently in the femoral vein, and there was no evidence of its having commenced higher up. During the latter days of the girl's life she was emaciated in the extreme, and it was impossible to collect the urinary secretion, or arrive at any details as to its quantity. But during the whole earlier periods of her ailments, and until what he had described as the last accident occurred, there was no albumen in the urine; and when the œdema suddenly developed she became unconscious and stupid, with occasional waking up and screaming, which phenomena he attributed to uræmic poisoning. There was no trace in the liver or spleen of any amyloid degeneration. The intestines were not examined for it, but there was nothing to lead them to suspect the existence of amyloid degeneration there either. The suddenness of the final attack of œdema drew his attention to the renal conditions; and then he examined such sample of the urine as he could get and found it albuminous. He felt justified in concluding that the fresh implication of the renal veins was the immediate cause of death; a week or so of it was, it seemed, sufficient to kill.

The Section then adjourned.

